# Left atrial thrombus formation within a few days of hospitalization in semi-acute ischemic heart disease despite no atrial fibrillation and mitral stenosis: a case report

**DOI:** 10.1186/s40981-020-00390-z

**Published:** 2020-10-24

**Authors:** Keiko Okamura, Mitsuharu Kodaka, Junko Ichikawa, Kazuyoshi Ando, Makiko Komori

**Affiliations:** Department of Anesthesiology & Intensive Care, Tokyo Women’s Medical University Medical Center East, Arakawa-ku, Tokyo, 116-8567 Japan

**Keywords:** Left atrial thrombus, Sinus rhythm, Activated partial thromboplastin time, Heparinization

## Abstract

**Background:**

Currently, the occurrence of left atrial thrombus despite the provision of heparinization within a few days of hospitalization without atrial fibrillation (AF) and mitral stenosis (MS) is rarely reported.

**Case presentation:**

A 71-year-old woman presented with chest discomfort and dyspnea. Examination revealed ST elevation with sinus rhythm, congestive heart failure, and moderate mitral regurgitation (MR) by transthoracic echocardiography (TTE). Diuretics, a coronary vasodilator, and unfractionated heparin (15,000 units/day) were administered. Four days after hospitalization, her C-reactive protein level had increased; therefore, TTE was repeated, revealing a thrombus in the left atrial appendage, which was probably affected by heparin resistance because of low antithrombin (49%). On day 5, the patient underwent emergency removal of the thrombus, mitral valve replacement, and coronary artery bypass.

**Conclusion:**

Patients can exhibit low left ventricular contractility, even sinus rhythm without MS. Thus, TTE and subsequent coagulation tests including antithrombin must be performed to prevent thrombus.

## Background

Left atrial thrombus (LAT) formation in patients with atrial fibrillation (AF) is potentially fatal because systemic embolization may occur. Anticoagulation therapy using warfarin or a direct oral anticoagulant is therefore mandatory to prevent embolism. Mitral stenosis (MS) is also a high-risk factor for LAT formation [1]. Conversely, mitral regurgitation (MR) slightly inhibits thrombus [2]. We herein describe a patient who was admitted to our institution for subacute myocardial infarction and congestive heart failure. She developed an approximately 2 cm LAT within 4 days of hospitalization without AF and MS despite undergoing anticoagulant therapy, heparinization, and MR.

## Case description

**Fig. 1:**
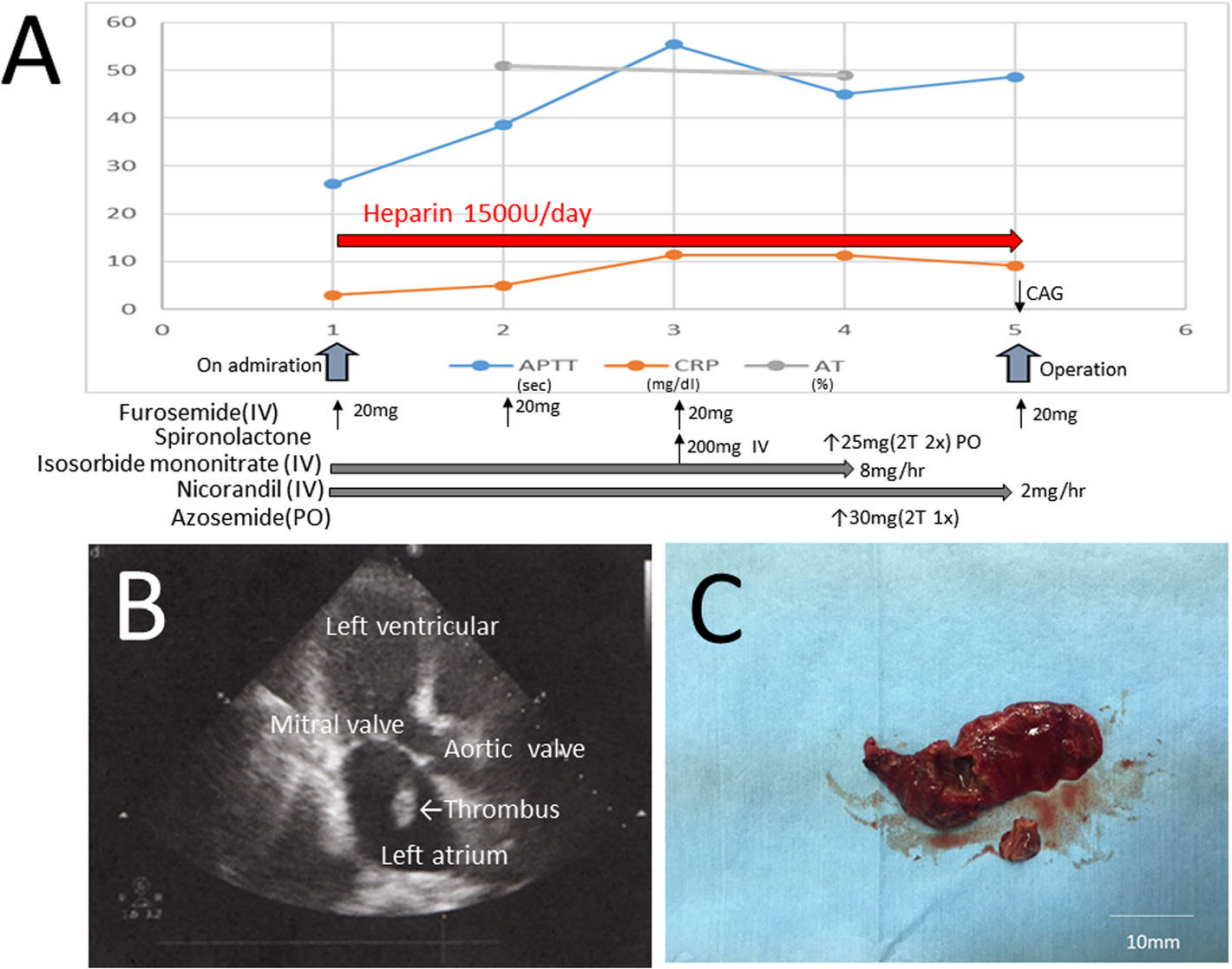
**a** Trends of activated partial thromboplastin time (APTT), CRP (C-reactive protein level), AT (antithrombin), heparinization, and other medications before surgery (*x*-axis, days; *y*-axis, measurement values). CAG coronary angiography, IV intravenous, PO per os. **b** Two-dimensional TTE immediately before surgery. **c** Removed thrombus attached at the left atrial appendage

A 52-kg, 71-year-old woman developed a slight fever (37.2 °C), chest discomfort, and dyspnea 2 weeks before hospitalization. Her external jugular veins were distended, her legs were edematous, and her oxygen saturation was low (88%) in room air. An electrocardiogram revealed ST elevation and a QS pattern in leads V1–4 without reciprocal change. Transthoracic echocardiography (TTE) revealed akinesis of the anterior septum, thinning (6 mm), a low ejection fraction (43%), hypokinesis at the interventricular apex, a dilated left atrium (50 mm), and mild tricuspid regurgitation peak pressure gradient (TRPG); 43 mmHg and moderate MR without MS. Her abnormal laboratory data were as follows: C-reactive protein (CRP), 2.95 mg/dl; alanine aminotransferase, 57 IU/l; gamma-glutamyl transferase, 64 IU/l; creatine phosphokinase-MB, 30 IU/l; Troponin T, 1.040 ng/mL; creatinine, 0.83 mg/dl; white blood cells, 10,000/μl; prothrombin time–international normalized ratio (PT-INR), 1.19; activated partial thromboplastin time (APTT), 26.2 s; D-dimers, 11.5 μg/ml; and N-terminal pro-brain natriuretic peptide, 11,265 pg/ml. These findings suggested liver congestion, renal dysfunction, and semi-acute myocardial infarction of the anterior septum. Cardiologists followed-up the ECG and cardiac enzyme 3 h later and found that the myocardial infarction had already peaked; they diagnosed that ST elevations with QS pattern misleadingly occurred because of semi-acute ischemia [3], suggesting that urgent coronary angiography (CAG) was unnecessary. Therefore, they decided to treat congestive heart failure first and avoided CAG to prevent further kidney injury (creatinine; 0.83 mg/dl). Diuretics (20 mg furosemide + 200 mg spironolactone/day), a coronary vasodilator (nicorandil + isosorbide mononitrate), and anticoagulant therapy (heparin at 15,000 U/day) were administered after hospitalization, resulted in an increase of APTT (Fig. 1a). Consequently, her symptoms improved. The TRPG decreased from 43 to 24 mmHg, the MR changed from moderate to mild, and the E/A improved from “restrictive to impaired” on day 3 in the cardiac care unit.

Four days after hospitalization, her CRP concentration was increased again to 11 mg/dl, indicating an inflammatory response possibly indicating infectious endocarditis (IE), and her antithrombin and fibrinogen levels were 49% and 420 mg/dl. Another TTE examination revealed a thrombus (24 × 14 mm) in the left atrial appendage (LAA) (Fig. 1b), moderate MR, and increased TRPG (40 mmHg). IE was first suspected, and emergency cardiac catheterization performed on day 5 of hospitalization indicated left anterior descending coronary artery #6, 90%; #7, 50–75%; and #9, 90%. Therefore, the patient had to undergo an emergency operation (thrombus removal, mitral valve replacement (MVR), coronary bypass, and LAA closure) despite having an uncontrolled heart failure.

General anesthesia was induced using fentanyl (0.2 mg), midazolam (3 mg), and rocuronium bromide (0.6 mg/kg), with nitroglycerin and nicorandil infusion. Intraoperative anesthesia was maintained by intravenously infusing remifentanil (0.2–0.5 μg/kg/min) and/or fentanyl with sevoflurane (1–3%) and/or propofol (2–3 μg/ml) with target-controlled infusion (Diprifusor™; Terumo, Tokyo, Japan). Furthermore, the radial arterial line, bispectral index (Nihon Kohden, Tokyo, Japan), regional SO_2_ (INVOS 5100C™; Edwards Lifesciences, Unterschleissheim, Germany), transesophageal echocardiography (TEE), central venous pressure, pulmonary artery pressure, continuous cardiac output, and SvO_2_ (Vigilance II™; Edwards Lifesciences) were considered for routine monitoring. A thrombus was attached to the LAA as confirmed by three-dimensional TEE (Video 1). The operative procedure comprised thrombus removal with LAA closure (Fig. 1c), MVR with a 27-mm biological valve (St. Jude Medical, Saint Paul, MN, USA), and coronary artery bypass grafting (aorta–saphenous vein–left anterior descending artery). The cardiopulmonary bypass was weaned without any untoward effects, and dopamine and milrinone were infused without any transvalvular leakage and other problems. The patient was discharged on the 17th postoperative day with no complications. Pathological examination of the excised mitral valve revealed fibrinolysis, hyalinosis, and mucoid degeneration without vegetative endocarditis. The thrombus consisted of the blood clot with fibrin, with no solid organization. In PAS, Grocott, and Gram staining, no bacterial colonies were detected on thrombus cultivation.


Additional file 1: **Video 1.** Three-dimensional TEE during surgery with sinus rhythm.

## Discussion

The patient manifested congestive heart failure because of semi-acute ischemic heart disease and moderate mitral regurgitation without any stenosis, but her electrocardiogram showed sinus rhythm without AF including past history. Anticoagulation therapy (heparinization) was administered, resulting to target 1.5 to 2.0 times [4] compared with control, and adequate prolongation of the APTT was achieved as indicated in Fig. 1a. The cardiologist initially suspected that the MR had resulted from IE because of the increased CRP level and slight fever. However, pathologic examination revealed that the correct diagnosis was fresh thrombus, not vegetation formation by infectious endocarditis, in spite of sinus rhythm. Notably, a thrombus had appeared in the patient’s LAA by 4 days after admission. A thrombus was less likely to be formed within 4 days, even with the patient’s extremely low contractility. The cardiologists specified two possible causes. First, the thrombus was already present in the LAA wall before admission because of the low left ventricular contractility or paroxysmal atrial fibrillation (PAF) and had then suddenly detached after insufficient heparinization. Second, the low antithrombin level (< 50%) induced heparin resistance, which could enlarge the root of the thrombus immediately within a few days.

The cause of LAT has also been described in two references. Matsuo et al. [2] reported that 4% of cases of LAT develop in patients with sinus rhythm, and one reason for this phenomenon is reduced cardiac contractility [2, 5] with or without AF. A dilated left atrium is also a risk factor for blood stasis. Additionally, the diagnostic power of TTE for LAT is lower than that of TEE (approximately 50% vs. 100%, respectively) [6].

The most common causes of LAT are severe rheumatic MS [1], AF, and congestive heart failure with an E/A of < 1 or lack of atrial kick [7]. In patients with a reduced left ventricular ejection fraction, i.e., 40% EF, both the dilated cardiac chambers, i.e., 50 mm left atrium and the impaired systolic function cause stasis of the blood within the heart, particularly the left atrium. Abnormalities in the blood flow are more common in areas of cardiac dyskinesis and severe systolic dysfunction. Flow abnormalities in patients with heart failure might be the major component of Virchow’s triad for thrombogenesis [8]. A limitation of our strategies was our failure to measure the antithrombin upon admission. It was immediately compensated by heparinization to avoid thrombus formation.

In conclusion, this report has described a rare case of semi-acute ischemic disease in a patient with left atrial thrombus without AF while undergoing heparinization. In patients with moderate MR and low left ventricular contractility without MS, even in the presence of sinus rhythm, TEE, and subsequent coagulation tests including APTT and antithrombin must be performed to prevent thrombus formation.

## Data Availability

Not applicable

## Abbreviations

AF: Atrial fibrillation
APTT: Activated partial thromboplastin time
CRP: C-reactive protein level
ECG: Electrocardiogram
IE: Infectious endocarditis
LAA: Left atrial appendage
LAT: Left atrial thrombus
MR: Mitral regurgitation
MS: Mitral stenosis
MVR: Mitral valve replacement
TEE: Transesophageal echocardiography
TTE: Transthoracic echocardiography
